# So You've Made a Novel Discovery: The History of Our Knowledge of Central Nervous System Lymphatics May Change Your Mind

**DOI:** 10.1055/a-2775-6770

**Published:** 2026-01-30

**Authors:** Peter C. Neligan

**Affiliations:** 1 Department of Surgery, University of Washington, Seattle, Washington, United States

**Keywords:** CNS lymphatics, central nervous system lymphatics, lymphatics

## Introduction

**Figure:**
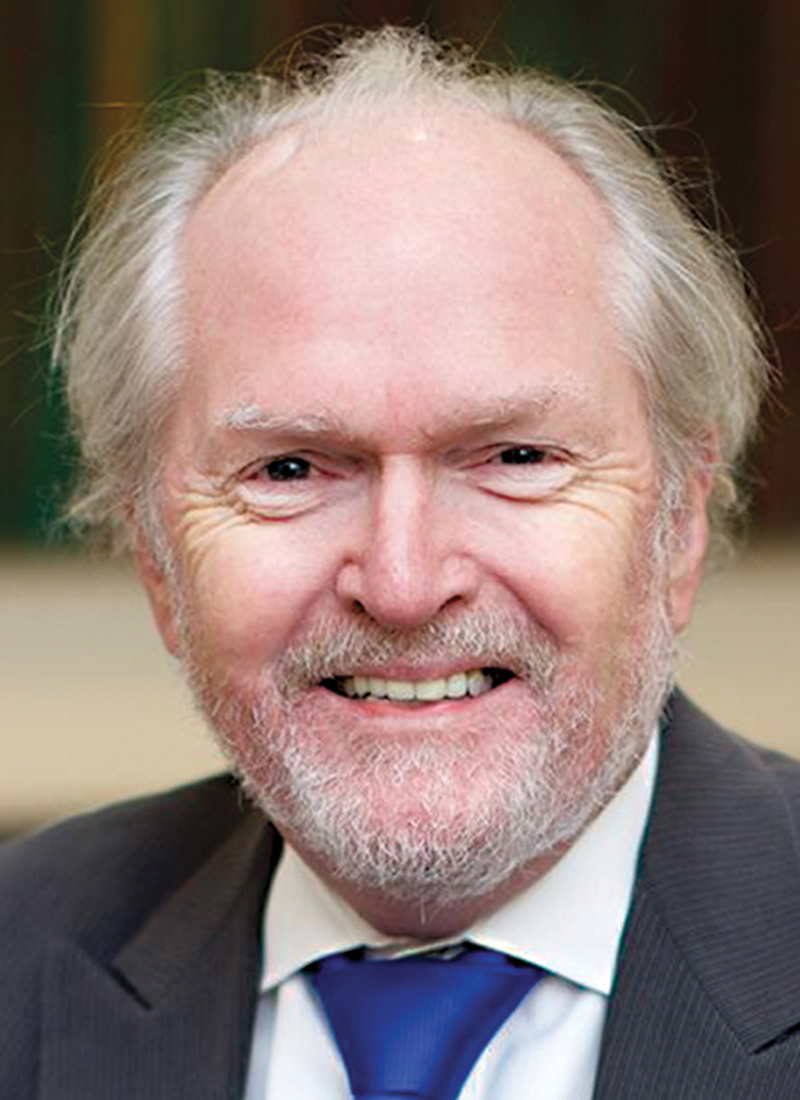
Peter C. Neligan

The lymphatic system is an organ system that is part of the immune system, which complements the circulatory system. It is, in fact, a parallel circulatory system that removes solutes from the interstitial fluid that are not reabsorbed by the postcapillary venules. Interstitial fluid consists of fluid that leaks from the vascular system, carrying nutrients to the cells and collecting waste products, bacteria, and damaged cells, before draining into the lymphatic vessels as lymph. The lymph drains to lymph nodes, which filter out unwanted constituents such as bacteria, cellular debris, etc.

## History of Central Nervous System Lymphatics

The brain has presented a conundrum in that the teaching has been that there are no identifiable lymph vessels in the brain. However, in 2012, Iliff et al described the glymphatic system. 1 This was a breakthrough because it explained how the brain could move solutes from its interstitium. It has long been thought that the cerebrospinal fluid (CSF) plays a role in solute clearance from the brain. 2 CSF, which is formed in the choroid plexi, flows through the cerebral ventricles and the subarachnoid space to its ultimate sites of reabsorption into the bloodstream via arachnoid villi of the dural sinuses, along cranial nerve sheaths, or through the nasal lymphatics. 3 4 5 Iliff et al demonstrated an influx route that ran along paravascular spaces surrounding penetrating arteries and an efflux route along paravenous spaces. They showed how solutes from the brain interstitium move in the interstitial fluid (ISF) from the parenchyma to the CSF, and that fluid flow between these anatomical influx and efflux routes is supported by astrocytic water transport. They called this the glymphatic system. So, for the first time, we were given a mechanism by which the brain could clear solutes through the interstitium.

We did not, however, have a clear understanding of what happened from there. However, in 2015, two separate publications demonstrated lymphatic vessels in the meninges of the mouse. 6 7 These lymphatic vessels were found lining the dural sinuses. Louveau et al surmised that “The unique location of these vessels may have impeded their discovery to date.” 7 In fact, the first report of meningeal lymphatics in the literature was in 1787 by Mascagni. 8 9 Paolo Mascagni was an Italian anatomist, and his findings were published in Latin and apparently never translated. It is thought that this may be a reason why his work was not widely read. His work was overshadowed by a publication by a Swedish anatomist, Magnus Gustaf Retzius and Axel Key, a German pathologist. It was published in German and gave a detailed description of the lack of lymphatic circulation in the brain. 10 This publication misled scientific thinking for 150 years. 11 Lukić et al reviewed the wax models that Mascagni made for the Josephinium in Vienna and wrote that “Mascagni could have been seduced by the appearance of the arachnoid, whose thread-like structures resemble lymph vessels. Also, he could have extended his finding of lymphatic drainage in every body organ to the conclusion that the brain should not be an exception.” 12 In the 38th edition of Gray's Anatomy, it is concluded that “Mascagni was probably so impressed with the lymphatic system that he saw lymph vessels even where they did not exist—in the brain.” 13

Sandrone et al have documented several other discoveries that have been made over the last century that were either forgotten or ignored. 11 Even before that, Schwalbe noted filling of the lymph vessels and nodes of the neck following injection of Berlin Blue into the subarachnoid space. 14 Many researchers over the last 150 years investigated how the subarachnoid space communicated with the lymphatics. It was generally believed that the subarachnoid fluid drained along the perineural spaces of the olfactory nerves to the tissue spaces of the nasal mucosa and from there, via fine lymphatic vessels, to the deep cervical lymph nodes. Brierley and Field showed this in rabbits in 1948, using subarachnoid India Ink injections, but also postulated that the deep cervical lymphatics and nodes filled through vessels coming through the jugular foramen. 15 In 1953, Lecco found lymphatics in the dura of four subjects. 16 In 1968, Földi et al described lymphatic structures in cerebral tissue from dogs and rats. 17 In 1987, Andres et al detected lymphatic structures within the dura of rats. He described how they leave the cranial cavity through the openings of the cribriform plate, rostral to the bulla tympani, together with the transverse sinus, and the middle meningeal artery. 18 Bradbury et al, in 1981, demonstrated drainage of cerebral interstitial fluid into the deep cervical lymph nodes of the rabbit. 19 In 1996, Li et al described meningeal stomata, which they believed were part of the cerebral prelymphatic capillary system, which undertakes cerebral lymph drainage. They noted that there were no lymphatic vessels in the brain, yet there is lymph drainage. 20

What all these papers show is that there has been intense interest and extensive research into the lymphatic functioning of the brain for centuries, even though, as far as many people are concerned, these discoveries are new, and the earlier discoveries I have cited were either ignored, forgotten, or disregarded.

## Conclusion

Understanding how the body works has involved an unbelievable depth of research over the centuries. I have highlighted just one area, CNS lymphatics; however, our understanding of human anatomy and physiology is an ongoing endeavor that has been conducted for a very long time. New discoveries are made all the time… or are they? It is important not to forget history and to thoroughly search the literature, no matter how old or obscure. In 1905, George Santayana, a Spanish philosopher, essayist, poet, and novelist, published “The Life of Reason: The Phases of Human Progress.” 21 In that book, he makes the statement “Those who cannot remember the past are condemned to repeat it.” It is so true! Remember it the next time you contemplate writing a paper!
